# Prediction of Patients’ Response to Immune Checkpoint Inhibitors Using Fluorescence Lifetime Imaging of Lymphocytes

**DOI:** 10.3390/biomedicines14051049

**Published:** 2026-05-05

**Authors:** Diana V. Yuzhakova, Anastasia I. Kononova, Anna V. Izosimova, Margarita M. Sazhina, Varvara V. Dudenkova, Vadim V. Elagin, Pavel A. Bureev, Dmitry A. Kuzmin, Alena I. Gavrina, Irina S. Shumskaya, Sergey V. Gamaynov, Marina V. Shirmanova

**Affiliations:** 1 Institute of Experimental Oncology and Biomedical Technologies, Privolzhsky Research Medical University, 10/1 Minin and Pozharsky Sq., 603005 Nizhny Novgorod, Russia; kononovaa0961@gmail.com (A.I.K.); annizosimova@mail.ru (A.V.I.); margo.sazh@mail.ru (M.M.S.); orannge@mail.ru (V.V.D.); bureev.pavel34@gmail.com (P.A.B.); kuzmin_dmitrij03@mail.ru (D.A.K.); gavrina.alena@mail.ru (A.I.G.); shirmanovam@mail.ru (M.V.S.); 2 Institute of Biology and Biomedicine, Lobachevsky State University of Nizhny Novgorod, 23 Gagarin Ave., 603950 Nizhny Novgorod, Russia; 3 Scientific Center for Translational Medicine, Sirius University of Science and Technology, 1 Olympic Ave., 354340 Sirius, Russia; 4 Nizhny Novgorod Regional Oncologic Hospital, 11/1 Delovaya St., 603163 Nizhny Novgorod, Russia; medicanns@mail.ru (I.S.S.); gamajnovs@mail.ru (S.V.G.)

**Keywords:** immunotherapy, immune checkpoint inhibitors, FLIM, lymphocytes, predictors

## Abstract

**Background/Objectives:** Although immune checkpoint inhibitors (ICIs) have dramatically transformed the landscape of cancer treatment, a significant percentage of patients are resistant to therapy. Identifying biomarkers to accurately predict patient response prior to treatment remains one of the key challenges in this field. Our study aimed to develop a platform for personalized prediction of immunotherapy efficacy for melanoma using isolated lymphocytes and fluorescence lifetime imaging (FLIM) of their autofluorescence. **Methods**: At the first stage, the ability of FLIM of the autofluorescent coenzyme NAD(P)H to resolve cellular response to anti-CTLA-4 was tested on lymphocytes isolated from lymphatic nodes (LNs) of mice with the B16 melanoma model. Then, cellular metabolic shifts in response to treatment with anti-PD-1 or its combination with anti-CTLA-4 were assessed via FLIM of patients’ blood lymphocytes. Activation of T-cells and alterations in the expression of metabolic genes after the treatment in vitro were additionally evaluated. **Results**: Fluorescence decay parameters α_1_ (free NAD(P)H fraction) and τ_2_ (protein-bound NAD(P)H lifetime) were identified as sensitive markers of lymphocyte activation after the treatment with immune checkpoint inhibitors. Responsive samples exhibited an increase in these parameters, which was associated with metabolic reprogramming toward more active glycolysis, the TCA cycle, fatty acid oxidation (FAO) and synthesis, the pentose phosphate pathway (PPP) and mitochondrial biogenesis. Importantly, the in vitro response of primary lymphocytes isolated prior to treatment correlated well with subsequent clinical outcomes. **Conclusions**: The proposed platform represents a promising solution for patient-specific immunotherapeutic drug screening and personalized treatment optimization.

## 1. Introduction

The discovery of immune checkpoint inhibitors (ICIs) has dramatically transformed the landscape of cancer treatment, offering a powerful therapeutic strategy for a wide variety of malignancies. Rather than attacking the tumor directly, ICIs target inhibitory receptors, specifically cytotoxic T-lymphocyte-associated antigen–4 (CTLA–4) and programmed cell death protein–1 (PD–1), preventing cancer escape from immune surveillance [1,2]. By inducing durable antitumor responses even in advanced cases, ICIs have become a standard of care for melanoma and other cancer types. Nevertheless, a significant percentage of patients demonstrate resistance to ICI therapy as well as immune-related adverse drug reactions due to host immune factors [3,4]. Identifying biomarkers to accurately predict patient sensitivity prior to or during early-stage treatment is one of the key challenges in modern oncology.

Currently, PD–L1 expression, tumor mutational burden (TMB) and microsatellite instability (MSI–H)/mismatch repair deficiency (MMRd) status are the primary clinically validated biomarkers for predicting ICI efficacy [5,6]. The value of PD–L1 is limited by predictive accuracy, intratumoral heterogeneity and assay variability; while TMB demonstrates a clinically modest correlation with response, high cost and time-consuming analysis [7,8], MSI–H/dMMR remains a robust tissue-agnostic marker with high predictive value in MSI–H tumors [9]. However, its clinical utility is restricted by its low occurrence in most solid tumors (2–4 % overall). Therefore, current clinical biomarkers remain suboptimal, highlighting the urgent need to identify more powerful predictors of patient response to ICIs. Since the host response to immunotherapy is a complex phenomenon mediated by various factors, comprehensive approaches are required. Strategies incorporating information from systemic factors such as the gut microbiome [10], tumor microenvironment [11] and circulating components [12,13] using multi-omics technologies combined with artificial intelligence [14] are currently under active consideration. However, their technical complexity and high costs currently limit their integration into routine clinical practice.

T-cell bioenergetic pathways are known as critical determinants of antitumor immunity [15]. Current reports indicate that PD–1 and CTLA–4 receptors compromise T-cell activation by repressing glycolysis and promoting fatty acid oxidation (FAO), while ICIs reverse metabolic exhaustion via the mTOR–Myc pathway, restoring glucose metabolism and IFN–γ production [16,17]. Since bioenergetic reprogramming precedes most functional shifts in immune cells such as changes in expression profile and proliferation, metabolic profiling of T-lymphocytes offers predictive potential for immunotherapy response.

Fluorescence lifetime imaging (FLIM) microscopy of cellular autofluorescence presents a non-invasive, label-free and highly sensitive technique which provides unique insights into cell metabolism [18,19]. Changes in fluorescence decay parameters of the reduced form of nicotinamide adenine dinucleotide (phosphate), NAD(P)H, deliver information about a range of metabolic perturbations, including alterations in bioenergetic and biosynthetic pathways [20], the activity of NAD(P)H-binding enzymes [21] and variations in the total NAD(H) pool size [22]. Recent studies reveal the utility of FLIM of NAD(P)H as a reliable tool for profiling immune cell metabolism [23,24]. We previously demonstrated that FLIM of NAD(P)H allows us to observe metabolic reprogramming of immune cells during tumor progression and immunotherapy in a mouse melanoma model [25,26]. This approach was further validated for predicting drug sensitivity in cellular patient-derived models [27,28].

In this study, we developed a cell-based in vitro platform for personalized prediction of ICI efficacy, utilizing primary lymphocytes and FLIM of NAD(P)H. The platform was established using the mouse B16F0 melanoma model, which remains the gold standard for testing melanoma immunotherapies [29,30,31], and validated using lymphocytes from melanoma patients’ blood collected prior to ICI therapy. Cellular metabolic shifts in response to in vitro anti-PD–1 or anti-CTLA–4 antibody treatment were assessed via NAD(P)H FLIM and verified by reverse transcription quantitative polymerase chain reaction (RT–qPCR) analysis of metabolic gene expression, as well as evaluation of T-cell activation by flow cytometry. Finally, the sensitivity of T-cells to ICIs as predicted in vitro was correlated with the subsequent clinical outcome.

## 2. Materials and Methods

### 2.1. Experimental Design

**Scheme 1:**
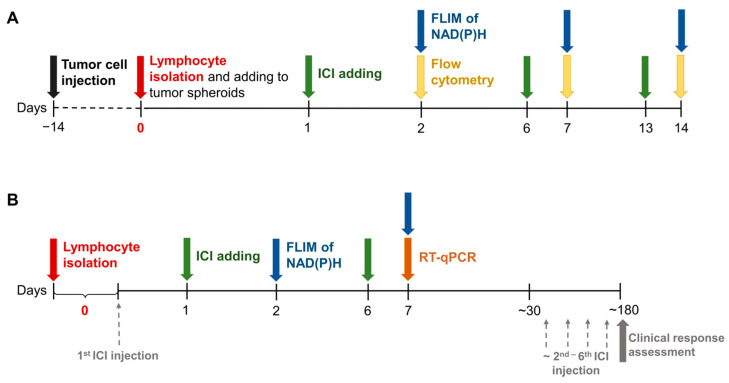
Experimental workflow for (**A**) the mouse tumor model and (**B**) patient samples.

The experimental workflows are demonstrated in Scheme 1.

In the animal experiments (Scheme 1A), at the first stage, the melanoma model B16F0 was established in mice (from day −14 to day 0). On day 0, lymphocytes were isolated from lymphatic nodes (LNs) and seeded into flat-bottom plates containing 1–2 B16F0 tumor spheroids per well as an additional source of antigen (the detailed procedure for spheroid preparation is provided in the Supplementary Materials). At the second stage (days 0 to 14), ICIs were added to the in vitro model on days 1, 6, and 13, and subsequently, flow cytometry and FLIM were performed on days 2, 7, and 14 (24 h post-treatment).

For the patient-derived samples (Scheme 1B), lymphocytes were isolated on day 0 from blood collected immediately prior to the first ICI injection into the patient. In vitro treatment was performed on days 1 and 6, followed by FLIM on days 2 and 7. RT–qPCR for metabolism-related gene expression was carried out on day 7. Clinical response data were obtained approximately 4–6 months (after ~4–6 treatment courses) following the initiation of therapy.

All studies on animals and patients’ material were approved by the Local Ethical Committee of the Privolzhsky Research Medical University (Nizhny Novgorod, Russia) (protocol No. 9 from 30 June 2023).

### 2.2. Mouse Tumor Model and LN Collection

A B16F0 melanoma model in C57Bl/6-FoxP3-EGFP mice (8 weeks old, females, weighing 20–22 g), kindly provided by A. Rudensky, Sloan Kettering Institute, New York, NY, USA [32], was used in the study. Mice were housed under standard conditions (12 h light/dark cycle, 22 ± 2 °C) with free access to food and water. Tumor cell culturing and implantation were performed as described in our previous studies [25,26]. Briefly, cells were expanded in RPMI-1640 medium (Roswell Park Memorial Institute 1640 medium) with L-glutamine and HEPES (Gibco, Amarillo, TX, USA). A total of 15 mice then received a subcutaneous injection of 1 × 10^5^ cells in 150 µL phosphate saline buffer (PBS) into the flank, close to the inguinal LN. Mice were anesthetized with Zoletil (40 mg/kg, 50 mL, Virbac SA, Carros, France) and 2% Xyla (10 mg/kg, 10 mL, Interchemie, Venray, the Netherlands) during the tumor cell injection.

The inclusion criterion was a palpable tumor; exclusion criteria were visible inflammation, injuries and poor body condition [33]. All mice were included in the study. Two weeks post-injection, mice were sacrificed by 90% isoflurane, and inguinal LNs were immediately micro-dissected via a median abdominal incision under sterile conditions.

### 2.3. Patient Blood Samples

Informed signed consent was obtained from all participants prior to their inclusion in the study. Peripheral blood samples were collected from 22 patients with diagnosed melanoma at the University Clinic (PRMU) who were scheduled for anti-PD–1 and/or anti-CTLA–4 immunotherapy. All patients were treatment-naïve, having received no prior chemotherapy or immunotherapy. No patients were excluded from the study. Detailed inclusion, non-inclusion and exclusion criteria are provided in the Supplementary Materials.

Peripheral blood (10 mL per patient) was collected immediately before the ICI injection, through the single-access catheter to minimize vascular damage, into ethylenediaminetetraacetic acid (EDTA)-coated vacuum tubes and maintained on ice during transport.

Patient characteristics are summarized in Table 1. The dataset included 22 individuals (9 males, 13 females) aged 40–80 years. Nineteen patients received anti-PD–1 monotherapy, while three received a combination of anti-PD–1 and anti-CTLA–4. Clinical efficacy was evaluated 4–6 months after the start of therapy using iRECIST criteria. An immune response (iR), including partial (iPR) or complete (iCR) responses, was observed in 10 patients; the remaining cases were classified as stable disease (iSD, *n* = 5) or progressive disease (iPD, *n* = 7).

### 2.4. Lymphocyte Isolation and Culturing

Mouse lymphocytes were isolated from freshly excised LNs by disaggregation with microsurgical scissors, followed by incubation in 1–2 mL of RPMI–1640 medium supplemented with 417 μg/mL Liberase TL (Roche, Mannheim, Germany) and 10 μg/mL DNase I (Roche, Mannheim, Germany) for 30 min at 37 °C on a shaker (80 rpm). After dissociation, the suspension was passed through a 70 μm strainer and washed twice with phosphate buffered saline (PBS).

Human lymphocytes were isolated from peripheral blood using Histopaque–1077 (Sigma-Aldrich, Stockholm, Sweden) according to the manufacturer’s instructions. Detailed isolation procedures for both models are described elsewhere [27].

Lymphocytes were seeded into 48-well flat-bottom plates at a concentration of 5 × 10^5^ cells (mice) and 2 × 10^5^ cells (human) (500 mL) per well in RPMI–1640 medium (with L–glutamine and HEPES) supplemented with 10% fetal bovine serum (FBS), 0.1% penicillin–streptomycin (Gibco, Amarillo, TX, USA) and 0.1% mouse or human interleukin–2 (Sigma-Aldrich, St. Louis, MO, USA). Two to three wells per group (untreated control versus ICI treatment) were used for FLIM microscopy (days 2, 7, and 14 for mice; days 2 and 7 for patients). For the mouse model, the same wells were subsequently used for flow cytometry. For patient lymphocytes, separate wells were used for RT–qPCR analysis on day 7.

The final immune cell fraction primarily consisted of CD3+ T-cells (≥68.4% for mouse and ≥72.4% for human samples on day 2), with their proportion notably increasing during cultivation. For patient samples, flow cytometry verified that CD19+ B-cells (≤8.2 or 5.1% on days 2 and 7, respectively) and the non-T/non-B-cell fraction (CD3−/CD19−) (≤13.8 or 7.4%) constituted a minor portion, as detailed in the Supplementary Materials (Supplementary Table S1).

### 2.5. In Vitro ICI Treatment

Mouse lymphocytes were treated with a therapeutic anti-CTLA–4 antibody (clone 9D9, Bio X Cell, Lebanon, NH, USA) at a final concentration of 8.5 µg/mL on days 1, 6, and 13. Patient-derived lymphocytes received the specific ICI treatment assigned for their clinical therapy: either anti-PD–1 pembrolizumab (Keytruda) (MSD International GmbH, Carlow, Ireland) or a combination of pembrolizumab and anti-CTLA–4 ipilimumab (Yervoy) (Bristol-Myers Squibb, Princeton, NJ, USA). Both human antibodies were added at a final concentration of 5 µg/mL on days 1 and 6.

### 2.6. FLIM of NAD(P)H

Immune cells were collected, centrifuged (300× *g*, 5 min), and resuspended in 50 µL of phenol red-free FluoroLite™ DMEM (Dulbecco’s Modified Eagle’s Medium) (Gibco, Amarillo, TX, USA). For imaging, cells were transferred to a 96-well black/clear plate (Falcon^®^, Corning, Berlin, Germany) and incubated for 5–10 min to allow sedimentation.

The detailed protocol of FLIM of NAD(P)H is described elsewhere [25,27] (Supplementary Materials). In brief, NAD(P)H microscopy (750 nm excitation; 450–490 nm detection) was performed on a laser scanning microscope, Zeiss LSM 880 (Carl Zeiss, Jena, Germany), equipped with a femtosecond laser (Spectra-Physics, Milpitas, CA, USA) and Time-Correlated Single Photon Counting setup (SPC–150N card, HPM–100–40 hybrid detector) (Becker & Hickl GmbH, Berlin, Germany).

FLIM data were analyzed using SPCImage 8.3 (Becker & Hickl GmbH, Berlin, Germany).

The following parameters were quantified: short (τ_1_) and long (τ_2_) lifetime components (free and protein-bound forms of the coenzyme, respectively) and their relative contributions (α_1_ and α_2_, α_1_ + α_2_ = 100%). The weighted average (mean) fluorescence lifetime was defined as τ_m_ = (α_1_ × τ_1_ + α_2_ × τ_2_)/(α_1_ + α_2_). For each sample, 30–40 morphologically identified lymphocytes were analyzed across 2–3 fields of view. The high purity of the T-cell population confirmed by flow cytometry and confocal fluorescence microscopy with CD3 and CD19 staining (see Supplementary Table S1 and Figure S1) ensured that the measurements predominantly represent T-cell responses.

### 2.7. Flow Cytometry

T-cell activation was evaluated in mouse lymphocytes by immunofluorescent labeling with antibodies to CD3, CD8, CD4, CD25, and CD69 (BD Biosciences, San Jose, CA, USA). The gating strategy was as follows: within the CD3+CD4+ population, regulatory (CD4+FoxP3+) and conventional (CD4+FoxP3–) T-cells were distinguished based on EGFP fluorescence, utilizing a transgenic C57BL/6 mouse model expressing an EGFP–FoxP3 chimeric construct. Both CD3+CD4+FoxP3– and CD3+CD8+ T-cells were further analyzed for the expression of activation markers CD69 and CD25. Data were collected using a BD FACSAria III cell sorter (BD Biosciences, San Jose, CA, USA) and processed in FlowJo v10.5.3 (BD/Treestar, Ashland, OR, USA), following the established protocol [25].

### 2.8. RT-qPCR

To evaluate metabolism-related gene expression, total RNA was extracted from patient lymphocytes using the HiPure Universal RNA Kit (Magen, Guangzhou, China). The samples were subsequently treated with DNase (Biolabmix, Novosibirsk, Russia) to remove residual genomic DNA. Reverse transcription was then performed using the reverse transcriptase kit “BioMaster RNAscribe RT Plus” (Biolabmix, Novosibirsk, Russia) to synthesize cDNA. Real-time PCR was conducted on a Bio-Rad CFX96 thermocycler (Bio-Rad, Hercules, CA, USA) using a SYBR Green-based reaction mixture (Invitrogen by Thermo Fisher Scientific, Carlsbad, CA, USA). The PCR reaction mixture contained 10X PCR buffer (Biolabmix, Novosibirsk, Russia), 250 μM of each dNTP, 0.5 nM of each primer, and 1 unit of HS Taq polymerase (Evrogen, Moscow, Russia). The final concentration of Mg^2+^ in the reaction was 3 mM.

The primer sequences are presented in Supplementary Table S2.

The thermal cycling conditions were as follows: initial denaturation and polymerase activation: 95 °C for 10 min; amplification (40 cycles): 95 °C for 15 s, 60 °C for 30 s, and 72 °C for 30 s; hybridization: 95 °C for 1 min and 40 °C for 1 min; melting curve analysis: 60 °C to 95 °C.

Reaction efficiency was determined using a standard curve. For data normalization, the reference genes POLR2A and ELF1 were used. Their stability was confirmed using CFX Maestro software (version 2.3) (Bio-Rad, Hercules, CA, USA). All qPCR reactions were performed in at least duplicate.

The data visualization was performed in GraphPad (version 8.0.1) (GraphPad, San Diego, CA, USA) via a heatmap. The gene expression was shown as log_2_ fold-change relative to control.

### 2.9. Statistical Analysis

The normality of distribution was evaluated using the Shapiro–Wilk test (at *p* ≥ 0.05, the distribution was considered normal). Differences between groups were analyzed using the Student’s *t*-test or Mann–Whitney U test in GraphPad Prism (version 8.0.1) software (*p*-value ≤ 0.05 was considered statistically significant), as indicated in the respective figure legends; data are presented as means ± SEM.

## 3. Results

### 3.1. Effects of Anti-CTLA-4 Treatment on Mouse Lymphocytes

#### 3.1.1. T-Cell Activation

To evaluate the in vitro response of mouse lymphocytes to anti-CTLA-4 treatment, we assessed the expression of early (CD69) and middle (CD25 (IL-2Rα)) activation markers in two main effector subsets, conventional CD4+ Th and CD8+ T-lymphocytes, on days 2, 7 and 14 (Supplementary Table S3). Since individual in vivo treatment outcomes could not be monitored in euthanized mice, analysis of T-cell activation by flow cytometry was used as a standard reference method to validate the cellular response.

**Figure 1:**
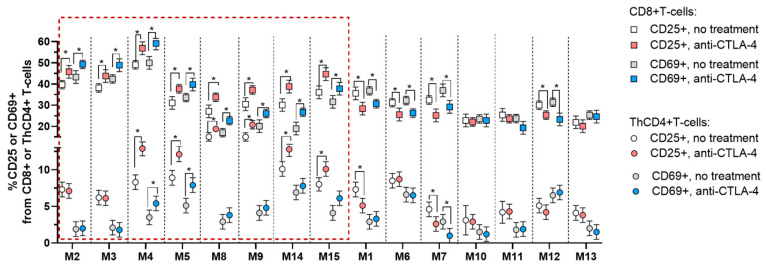
Expression of the activation markers CD25 or CD69 in CD8+ or CD4+ mouse T-lymphocytes on day 14 of culturing in the presence of anti-CTLA-4. Dot plots show measurements for individual mice (dots) and SEM (horizontal lines). The red box indicates the cases of a significant rise in the percentage of CD25+ or CD69+ cells, either in the CD8+ T-cell subset or in both CD8+ and CD4+ T-cells. Statistics: Mann–Whitney U test. * Significant difference, *p* ≤ 0.05.

The most significant changes in the percentage of activated CD25+ or CD69+ cells compared to the untreated control were observed on day 14 (Figure 1).

After anti-CTLA-4 treatment, 8 out of the 15 samples (M2, M3, M4, M5, M8, M9, M14 and M15) demonstrated a significant increase in the percentages of CD25+ and CD69+ cells in the CD8+ T-cell subset in comparison with the untreated controls. Furthermore, the samples M4, M5, M8, M9, M14 and M15 exhibited a statistically significant increase also in the CD4+ Th cell subset in CD25+ cells (M8, M9, M14 and M15) or both CD25+ and CD69+ cells (M4 and M5). These cases were classified as an “activation response”.

The remaining 7 samples showed no changes (M10 and M11) or a significant decrease in the percentage of CD25+ and CD69+ cells in the CD8+ T-cell subset. Additionally, M1 and M7 had a decline in percentages of activated CD4+ Th cells. These cases were classified as “no activation response”.

#### 3.1.2. Autofluorescence Lifetime Parameters of NAD(P)H

The autofluorescence decay parameters of NAD(P)H were analyzed in the mouse lymphocytes on days 2, 7 and 14 of culturing in the presence of anti-CTLA-4 and compared with the corresponding untreated controls (Table 2 and Table S4).

To find the most sensitive parameter among the FLIM data to the activation of T-cells, correlation with the expression of the activation markers was performed.

Of the decay parameters, the relative contribution of free NAD(P)H a1 and the fluorescence lifetime of protein-bound NAD(P)H τ_2_ showed the best correspondence with the activation response.

In the group with “activation response” on day 2, five out of eight samples (M3, M4, M5, M9 and M14) demonstrated a statistically significant increase either in the relative contribution of free NAD(P)H α_1_ or the lifetime of protein-bound form τ_2_ or in both parameters (M3, M4, M5, M9 and M14) (for example, 69.1 ± 0.8 versus 72.2 ± 0.7%, *p* = 0.0008, and 2.95 ± 0.08 versus 3.66 ± 0.05 ns, *p* < 0.0001 for α_1_ and τ_2_, respectively, in M5) (Table 2 and Table S4). In the other three samples (M2, M8 and M15) the autofluorescence lifetime did not change.

**Figure 2:**
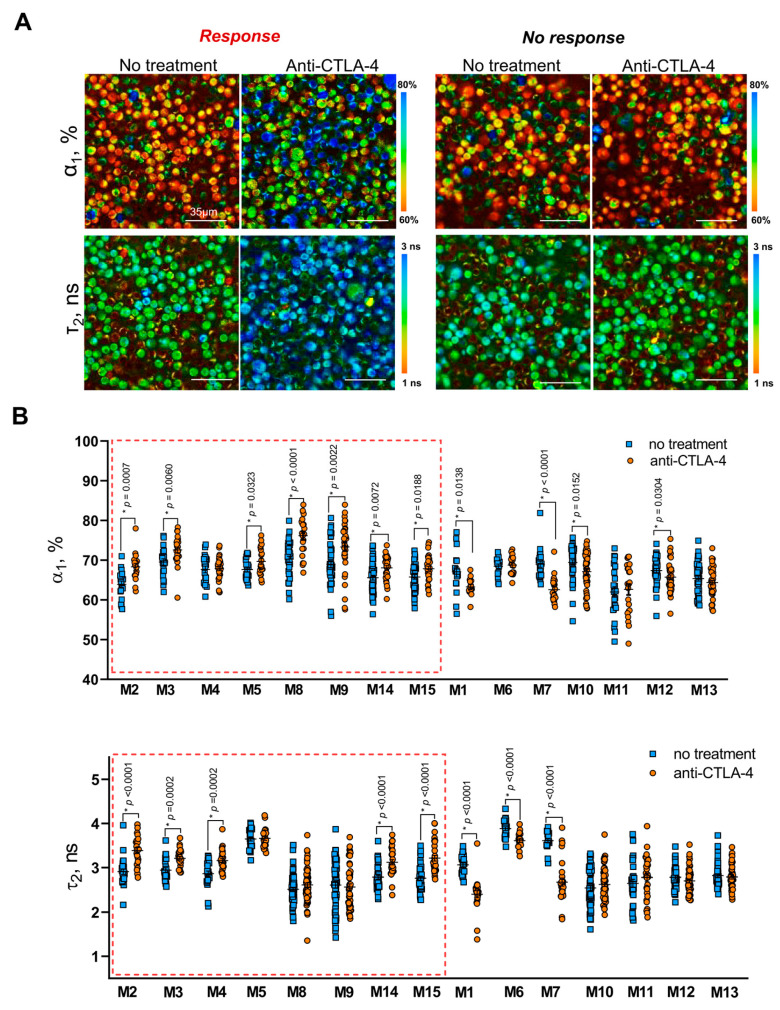
FLIM of NAD(P)H in the mouse lymphocytes on day 7 after in vitro anti-CTLA-4 treatment. (**A**) Representative FLIM images of lymphocytes of responsive (M2) and non-responsive (M11) samples. The relative amplitude of free NAD(P)H α_1_ and the fluorescence lifetime of protein-bound NAD(P)H τ_2_ are shown in the untreated and treated lymphocytes. The scale bar is indicated in the images. (**B**) Quantification of NAD(P)H α_1_ and τ_2_ for individual mouse samples. The graphs display the mean and SEM (horizontal lines). Dots are the measurements for individual cells. The red boxes indicate the cases of an activation response. Statistics: Student’s *t*-test. * Significant difference, *p* ≤ 0.05.

On days 7 and 14 all “activation responders” showed a significant increase in either α_1_ or τ_2_ or in both parameters compared to untreated controls (for example, 70.3 ± 0.9 versus 76.2 ± 0.8%, *p* < 0.0001 for α_1_ in M8 and 2.86 ± 0.06 versus 3.16 ± 0.04 ns, *p* = 0.0002 for τ_2_ in M4 on day 7) (Figure 2).

Concerning the group with “no activation response”, on day 2 the autofluorescence lifetime parameters did not change (M10, M11, M12 and M13) or showed a decrease in α1 or/and τ2 (M1, M6 and M7). On day 7, most non-responders (M1, M6, M7, M10 and M12) demonstrated a decline in one or both of the a1 and t2 parameters, while M11 and M12 remained unchanged. On day 14, all samples in this group exhibited a decrease in at least one or both parameters (for example, 67.7 ± 0.8 versus 63.4 ± 0.8%, *p* = 0.0163 and 2.96 ± 0.06 versus 2.20 ± 0.06 ns, *p* < 0.0001 for α_1_ and τ_2_, respectively, in M12).

The observed changes in autofluorescence lifetime parameters are most likely associated with a shift to glycolysis (α_1_) [20] and a modified profile of NAD(P)H-binding enzymes (τ_2_) [21] involved in both bioenergetic and biosynthetic processes. The first phenomenon is consistent with the restoration of glycolysis by anti-CTLA-4 therapy [16,17] and correlated with our previous studies on in vitro patient-derived [27] and in vivo mouse melanoma [25] models.

Changes in other fluorescence decay parameters (fluorescence lifetime of free NAD(P)H τ_1_ and the mean fluorescence lifetime τm) had worse correspondence with the activation profile (Supplementary Table S5). NAD(P)H τ_1_ increased in only five of 24 cases among the “response” samples at all time points (M3, M5, M14) and decreased in six of 21 cases among “non-responders” (M1, M3, M7, M12). NAD(P)H τ_m_ significantly increased in nine “response” cases and decreased in nine “no response” cases, primarily as a result of changes in τ_1_ and τ_2_, whereas in one “response” case it decreased due to an increase in α1.

Therefore, these results show that selected NAD(P)H fluorescence decay parameters, such as α_1_ and τ_2_, are sensitive markers of lymphocyte activation after anti-CTLA-4 treatment in vitro, resolving the heterogeneous response of isolated cells from different mice as early as day 7 of cultivation.

### 3.2. Effects of ICI Treatment on Patient Lymphocytes

#### 3.2.1. Patients’ Clinical Response to ICI Therapy

Clinical response assessment revealed a response in 10 cases, including partial or complete response to anti-PD-1 therapy (iPR, *n* = 6; iCR, *n* = 2) and complete response to a combination of anti-PD-1 and anti-CTLA-4 (iCR, *n* = 2) (Table 1). Stable disease (iSD) with anti-PD-1 therapy was observed in five cases, while progressive disease occurred in seven cases (iCPD, *n* = 5 and iUPD, *n* = 1 for anti-PD-1; iCPD, *n* = 1 for combination therapy).

The cellular response data obtained during in vitro corresponding ICI treatment (anti-PD-1 or a combination, where appropriate) were further correlated with the clinical outcomes to validate the predictive efficiency of the platform.

#### 3.2.2. FLIM of NAD(P)H

The autofluorescence decay parameters of NAD(P)H were analyzed in the patients’ lymphocytes on days 2 and 7 after treatment with anti-PD-1 or a combination of anti-PD-1 and anti-CTLA-4 and compared with the corresponding untreated controls (Table 3 and Table S6). The results of FLIM were then compared with further clinical outcomes.

In the group with “response” on day 2, eight out of ten patient samples demonstrated a statistically significant rise in α_1_ (P13) or τ_2_ (P2, P7, P15 and P18) or in both parameters (P12, P17 and P23) (for example, 71.4 ± 0.8 versus 75.8 ± 0.8%, *p* = 0.0003 and 3.32 ± 0.06 versus 3.86 ± 0.05 ns, *p* < 0.0001 for α_1_ and τ_2_, respectively, in P17) (Table 3 and Table S6). In the other two samples (P4 and P16), the autofluorescence lifetime did not change.

**Figure 3:**
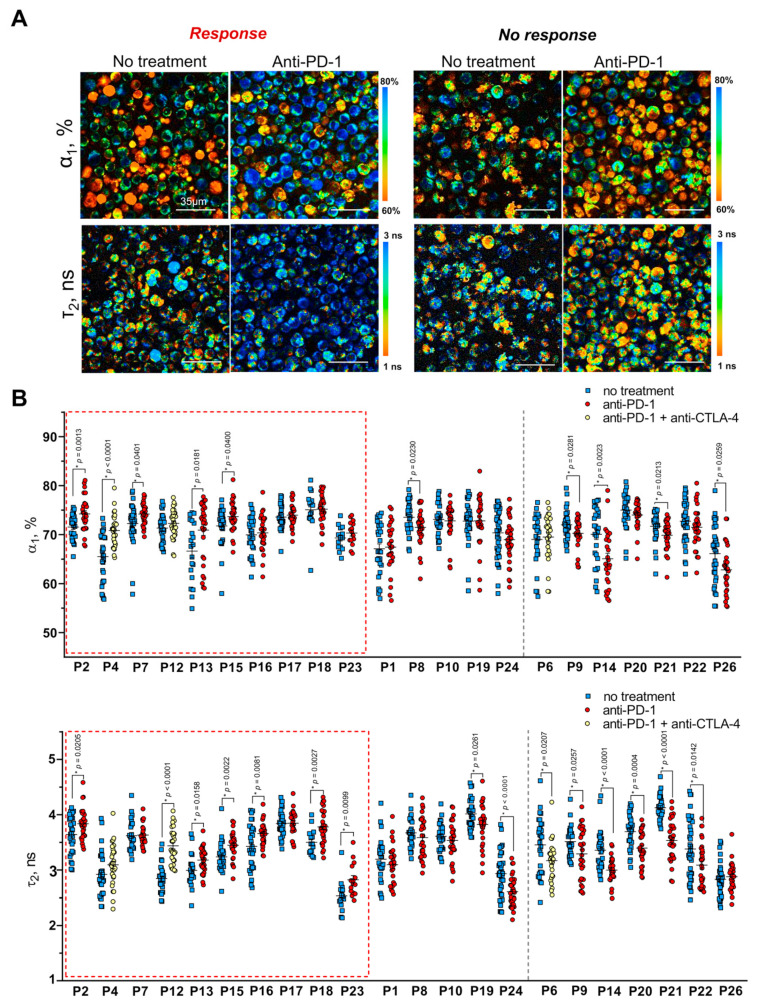
FLIM of NAD(P)H in the patient lymphocytes on day 7 after in vitro ICI anti-PD-1 or a combination of anti-PD-1 and anti-CTLA-4 treatment. (**A**) Representative FLIM images of lymphocytes of responsive (P2) and non-responsive (P14) samples. The relative amplitude of free NAD(P)H α_1_ and the fluorescence lifetime of protein-bound NAD(P)H τ_2_ are shown in the untreated and treated lymphocytes. The scale bar is indicated on the images. (**B**) Quantification of NAD(P)H α_1_ and τ_2_ for individual patient samples. The graphs display the mean and SEM (horizontal lines). Dots are the measurements for individual cells: untreated (blue), and treated with anti-PD-1 (red) or anti-PD-1 and anti-CTLA-4 (yellow). The red boxes indicate the cases of a clinical response; the grey dashed line separates cases of stable (**left**) and progressive (**right**) disease. Statistics: Student’s *t*-test. * Significant difference, *p* ≤ 0.05.

On day 7 all of the samples except P17 demonstrated an increase in either one (P4, P7, P12, P16, P18 and P23) or both (P2, P13 and P15) decay parameters (for example, 71.8 ± 0.7 versus 73.7 ± 0.6%, *p* = 0.04 and 3.22 ± 0.05 versus 3.45 ± 0.04 ns, *p* = 0.0022 for α_1_ and τ_2_, respectively, in P15) (Figure 3). In one sample (P17) α_1_ and τ_2_ did not differ from the control measurements; however, they maintained high baseline values (73.6 ± 0.5 versus 73.6 ± 0.6% and 3.84 ± 0.04 versus 3.86 ± 0.04 ns for α_1_ and τ_2_, respectively).

In the group with “stable disease”, on day 2, two out of five samples (P8 and P10) demonstrated a decrease in τ_2_, while one sample (P19) showed an increase. By day 7, three samples showed a decrease in either α_1_ (P8) or τ_2_ (P19 and P24), whereas two samples (P1 and P10) remained unchanged.

In the “progressive disease” group, four out of seven samples (P6, P9, P14, and P26) showed a significant decrease in one (P9, P14 and P26) or both (P6) decay parameters (P6, 73.3 ± 0.6 versus 70.4 ± 1.3%, *p* = 0.0088 and 3.59 ± 0.05 versus 3.11 ± 0.05 ns, *p* < 0.0001 for α_1_ and τ_2_, respectively). On day 7, all of the samples had a statistically decreased value of α_1_ or τ_2_ (P6, P20, P22 and P26) or both (P9, P14 and P21) (for example, 70.1 ± 0.0 versus 65.1 ± 1.0%, *p* = 0.0023 and 3.37 ± 0.07 versus 3.00 ± 0.07 ns, *p* < 0.0001 for P14).

Changes in other fluorescence decay parameters (τ_1_ and τm) had worse correspondence with the activation profile (Supplementary Table S7).

#### 3.2.3. Analysis of Metabolic Gene Expression

**Figure 4:**
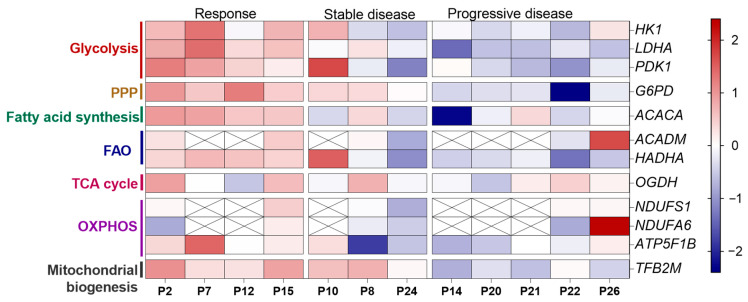
Heatmap demonstrating expression of the metabolism-related genes in patient lymphocytes on day 7 after in vitro ICI treatment. The color scale represents the log_2_ fold-change in gene expression: shades of red indicate upregulation, while shades of blue indicate downregulation in response to ICI treatment compared to the corresponding untreated control. The intensity of the color reflects the magnitude of the change, with white indicating non-significant changes. Crossed boxes indicate data unavailable due to insufficient RNA quality or quantity.

To clarify which metabolic pathways caused alterations in NAD(P)H fluorescence lifetimes of patient lymphocytes, the expression of key metabolic genes was analyzed in 12 samples, for which a sufficient amount of material was available. The analyzed genes included those involved in glycolysis (*HK1*, *LDHA*, *PDK1*), the pentose phosphate pathway (PPP) (*G6PD*), fatty acid synthesis (*ACACA*), FAO (*ACADM*, *HADHA*), the tricarboxylic acid (TCA) cycle (*OGDH*), oxidative phosphorylation (OXPHOS) (*NDUFS1*, *NDUFA6*, *ATP5F1B*), and mitochondrial biogenesis (*TFB2M*) (Figure 4).

Clinical responders were characterized by a significant increase in the expression of genes associated with glycolysis, PPP, fatty acid synthesis and oxidation, TCA cycle and mitochondrial biogenesis in lymphocytes (Figure 4). An increase in the expression of mitochondrial respiration genes was detected in particular cases (P7 and P15).

In contrast, the group of patients with “progressive disease” demonstrated downregulated expression of most metabolism-related genes. In the “stable disease” group, various patterns of gene expression were detected, ranging from a slight rise to divergent changes or a pronounced decrease in expression of genes related to energy metabolism and biosynthesis.

The observed differences in gene expression profiles between responsive and non-responsive patients suggest global metabolic reprogramming of T-cells upon their reactivation. In the process of glycolysis, FAO and the TCA cycle, NAD+ is reduced to NADH, resulting in an increase in the free NAD(P)H fraction (α_1_), which was detected in the lymphocytes of responders. PPP and fatty acid biosynthesis are associated with NADPH binding to glucose-6-phosphate dehydrogenase (G6PD), which provides a longer fluorescence lifetime for the coenzyme [21,34], which can explain the increase in the long lifetime τ_2_ in the lymphocytes.

## 4. Discussion

Over the past 30 years, the incidence of melanoma has increased more rapidly than any other malignancy, with approximately 330,000 new cases reported annually worldwide. Melanoma is traditionally recognized as a highly aggressive skin tumor with poor outcomes. The treatment landscape shifted dramatically in 2011 with the approval of the first ICI (ipilimumab) and the first BRAF-targeted therapy (vemurafenib) [35]. Prior to these breakthroughs, the 5-year survival rate for advanced melanoma was only approximately 6%, with a median overall survival of 7–8 months, while the current long-term survival rate is over 50%, with complete cures for previously fatal tumors. Despite the achieved progress, approximately 40–50% of melanoma patients exhibit resistance to immunotherapy, and furthermore, therapeutic efficacy is often limited by significant toxicity [36].

Clinically validated biomarkers for predicting ICI response include the following factors. Among them, BRAF V600 mutation status remains a key determinant for treatment stratification, guiding the choice between targeted therapy and immunotherapy in the first-line setting [37]. Regarding direct predictors of ICI response, PD–L1 expression on tumor cells (FDA-approved in 2015) remains the most widely used biomarker [5]. However, its clinical utility is significantly limited by assay variability and intratumoral heterogeneity. Notably, up to 40% of PD–L1-negative patients derive clinical benefit, whereas some tumors with high PD–L1 expression remain resistant to treatment. Enhanced predictive accuracy can be achieved by utilizing a multimodal biomarker panel. The high sensitivity of dMMR/MSI–H tumors to ICIs led to their FDA approval as a tissue-agnostic biomarker in 2017. However, while relatively frequent in endometrial and colorectal cancers, this marker is rarely observed in melanoma, with an incidence rate not exceeding 1–2% [9]. In contrast, TMB (FDA-approved in 2020) exhibits high levels in melanoma due to ultraviolet exposure and correlates more strongly with treatment response; nevertheless, inconsistent correlations across cancer histologies and a lack of standardized assays restrict its implementation as an agnostic predictive biomarker [38]. Therefore, current clinical biomarkers remain suboptimal, making the identification of new predictors one of the primary goals in the field of immunotherapy [39]. Testing immunotherapy efficacy using patient-specific cellular models containing patient-derived tumor and immune cells [40,41,42] represents an alternative approach.

The mechanisms of melanoma resistance to ICIs are diverse, ranging from the loss of wild-type antigen expression, disruption of antigen presentation and defects in IFNγ signaling to limited gut microbiome diversity and rearrangements in the immune cell profile [43,44]. Metabolic reprogramming of the immune microenvironment is one of the crucial factors, since melanoma development intensively involves metabolic pathways competing with effector cells [45]. Enhanced rates of glycolysis by activating the MAPK and PI3K/Akt/mTORC1 signaling pathways and high amino acid and lipid metabolism in melanoma cells lead to the formation of an unfavorable acidic microenvironment with lactate accumulation and glucose deprivation. Since activated T-cells themselves depend on upregulated glycolysis and glutaminolysis, the accumulation of lactate inhibits their proliferation and cytokine synthesis. This metabolic imbalance ultimately drives effector T-cell exhaustion while simultaneously promoting the stability and function of regulatory T-cells. Consequently, the metabolic profile of T-cells serves as a promising predictive biomarker for therapeutic response in melanoma patients.

FLIM of NAD(P)H and flavin coenzymes provides a non-invasive, label-free approach [46] for the assessment of the metabolic state of live immune cells [23], potentially overcoming the limitations of conventional methodologies. However, the potential of this approach in the context of personalized ICI response prediction has yet to be fully explored. Our previous research established that FLIM of NAD(P)H in ex vivo lymphoid tissues can effectively monitor immune dynamics during tumor development [25] and anti-CTLA–4 treatment [26] on a melanoma model. Most recently, by employing a patient-derived glioma model with an autologous immune component, we observed that the NAD(P)H decay profile correlates with antitumor activity of lymphocytes during ICI treatment, thereby validating its potential as a reliable immunotherapy drug-response biomarker [27].

In the current study, we developed a cellular platform for the personalized prediction of ICI efficacy. At the initial stage, a syngeneic mouse B16F0 melanoma model was used. It is important to note that the model demonstrated response heterogeneity even within a shared genetic background. This variability is likely driven by the stochasticity of the T-cell receptor repertoire (arising from Variable (Diversity) Joining (V(D)J) recombination) [47], resulting in varying numbers of tumor-specific T-cells, as well as individual differences in lymphocyte subpopulations (particularly the percentage of regulatory T-cells) and systemic pre-activation in vivo. Variable responses to ICI have also been reported in in vivo studies using other syngeneic models [48]. Since similar individual immune features are involved in the response of human lymphocytes [49,50], this model is relevant for translational studies.

The developed platform was further validated using blood samples from melanoma patients collected prior to therapy. We demonstrated that the therapy-induced alterations in NAD(P)H fluorescence lifetimes measured in patients’ peripheral blood lymphocytes correlated with their following clinical outcome. Specifically, responders exhibited an increase in one or two parameters of NAD(P)H autofluorescence α_1_ and τ_2_, whereas non-responders showed no changes or a marked decrease. The observed elevation of α_1_ in responders corresponds to a metabolic shift toward glycolysis, supporting the increased energy and biosynthetic requirements of effector T-cells. Metabolic reprogramming of T-cells was verified by RT–qPCR, showing the upregulation of glycolysis-associated genes. In turn, changes in τ_2_ may be attributed to alterations in the specific NAD(P)H-binding enzyme profile [21], particularly the enhanced activity of G6PD [34], PPP and following biosynthetic processes, consistent with our RT–qPCR data.

The suggested mechanism of action for ICIs in the developed in vitro platform involves several factors. First, the lymphocytes were pre-activated by the tumor in the organism prior to isolation. In patients, advanced melanoma is known to be highly immunogenic [51], ensuring a pool of primed T-cells; in the mouse model, this state was additionally maintained by the introduction of tumor spheroids. Second, the culture contains a minor population of CD80/86+ B-cells and monocytes, which can serve as antigen-presenting cells (APCs). Regarding anti-CTLA-4 treatment, blocking the corresponding receptor allows CD28 on T-cells to interact with CD80/86, resulting in a costimulatory signal. This mechanism may presumably expand the pool of tumor-specific effector cells [52]. Regarding anti-PD-1 treatment, the ICI blockade can restore the function of exhausted T-cells [53] and prevent the receptor from interacting with PD-L1 expressed by other cells in the culture, such as regulatory B-cells [54]. Moreover, Ganesan et al. demonstrated a significant increase in interleukin-2 and interferon-gamma production, as well as T-cell proliferation, in human peripheral blood mononuclear cells (PBMCs) treated with small molecule inhibitors or pembrolizumab without the addition of any exogenous APCs [55].

Our study has several limitations. First, the relatively modest cohort size, while providing proof-of-concept, necessitates further validation in larger, multi-center studies.

It should also be mentioned that the proposed platform is applicable to highly immunogenic tumors, such as melanoma. The extension of this technique to low-immunogenic malignancies might require the in vitro activation of immune cells through co-cultivation with a source of tumor antigens, for instance, tumor explants [27].

The third limitation is the use of laser scanning microscopy-based FLIM for NAD(P)H registration, which is a technically complex and expensive method. We used FLIM microscopy to precisely measure fluorescence lifetimes in the cytoplasm of individual cells and, thus, account for intercellular metabolic variability. Given that, in response to treatment, changes in the NAD(P)H lifetime parameters are observed in the majority of T-cells in a cell population in the responder group, microscopic resolution may be unnecessary for routine clinical screening.

In the future, more cost-effective and clinically relevant techniques, such as wide-field or confocal macro-FLIM [56] or fiber-optic-based systems, can be considered as an alternative to FLIM microscopy with a clearer perspective of adoption in routine clinical practice. When coupled with machine learning-based analytics, these innovations will facilitate rapid, high-throughput screening of immunotherapy drugs for individual patients.

## 5. Conclusions

In this study, we developed a cellular platform based on patient-derived lymphocytes for the personalized prediction of ICI efficacy. We demonstrated that FLIM of NAD(P)H autofluorescence effectively discriminates between responders and non-responders, with increases in two FLIM parameters (α_1_ and τ_2_) serving as sensitive predictors of favorable clinical outcomes. The metabolic shifts toward enhanced glycolysis and biosynthetic activity were further confirmed by RT-qPCR analysis of key metabolic genes. The proposed platform represents a promising solution for rapid, patient-specific immunotherapeutic drug screening and treatment optimization.

Future advancements involving more cost-effective alternatives to microscopy, such as wide-field macro-FLIM or fiber-optic systems coupled with machine learning analytics, will enable high-throughput drug screening, making the platform a financially accessible tool for routine clinical practice. Furthermore, the extension of this technique to low-immunogenic malignancies may involve enhancing the platform by co-cultivating immune cells with a source of tumor antigens, such as tumor explants, to achieve optimal in vitro activation.

## Figures and Tables

**Table 1:** Clinicopathological data of the patients.

Sample Code	Age	Sex	cTNM or pTNM Staging	Clinical Stage	BRAF Status	Therapy	Response (iRECIST)
P1	73	F	pT4bNxM1	IV	Wild type	Anti-PD-1	iSD
P2	80	F	cT1aN0M0	IA	Wild type	Anti-PD-1	iCR
P3	73	F	pT4bN0M0	IIB	Wild type	Anti-PD-1	iPD
P4	62	F	TxNxM1	IV	Wild type	Anti-PD-1+ Anti-CTLA-4	iCR
P6	60	M	pT2_B_N1a	IIIA	Wild type	Anti-PD-1+ Anti-CTLA-4	iCPD
P7	46	M	TxN2M1	IV	V600E	Anti-PD-1	iPR
P8	61	F	pT0N1M1a	IVA	Wild type	Anti-PD-1	iSD
P9	70	M	pT4bN0M0	IIC	V600E	Anti-PD-1	iCPD
P10	64	M	pT4bN0M0	IIC	V600E	Anti-PD-1	iSD
P12	74	F	cT2aN1M0	IIIA	V600E	Anti-PD-1+ Anti-CTLA-4	iCR
P13	70	M	pT4bN0M0	IIC	K601N	Anti-PD-1	iPR
P14	67	F	pT4_B_N0M0	IIC	Wild type	Anti-PD-1	iCPD
P15	62	F	pT2aN0M0	IB	Wild type	Anti-PD-1	iPR
P16	73	F	pT1bN0M0	IA	V600E	Anti-PD-1	iCR
P17	76	M	pT2bN1cM1	IV	V600K	Anti-PD-1	iPR
P18	73	F	pT1bN0M0	IA	V600E	Anti-PD-1	iPR
P19	76	M	pT4bN1cM1c	IVC	V600E	Anti-PD-1	iSD
P20	63	M	pT4bN0M0	IIC	V600E	Anti-PD-1	iUPD *
P21	72	F	pT2aN0M0	IB	Wild type	Anti-PD-1	iCPD
P22	61	M	pT4bN0snM0	IIC	V600K	Anti-PD-1	iCPD
P23	40	F	cT3bN0M0	IIB	Wild type	Anti-PD-1	iPR
P24	73	F	pT2aN0M0	IB	Wild type	Anti-PD-1	iSD
P26	80	F	cTxN0M1	IV	V600E	Anti-PD-1	iCPD

“M” or “F” indicates male or female, respectively; “cTNM or pTNM staging” indicates clinical or pathological Tumor–Node–Metastasis staging; “V600E”, “V600K” or “K601N” indicates BRAF mutation in exon 15; “iPR” or “iCR” indicates immune partial or complete response; “iSD” indicates immune stable disease; “iCPD” or “iUPD” indicates confirmed or unconfirmed progressive disease; “*” indicates death.

**Table 2:** Changes in autofluorescence lifetime parameters of NAD(P)H α_1_ and τ_2_ in the mouse lymphocytes on days 2, 7 and 14 after in vitro anti-CTLA-4 treatment in comparison with the corresponding untreated controls. The color of the square indicates, respectively, a statistically significant (*p* ≤ 0.05, Student’s *t*-test) increase (green) or decrease (red) in a fluorescence lifetime parameter value compared to the corresponding untreated control. The colorless squares indicate no significant difference.

	Sample Code	Day 2		Day 7		Day 14	
		α_1_	τ_2_	α_1_	τ_2_	α_1_	τ_2_
Activation response	M2						
	M3						
	M4						
	M5						
	M8						
	M9						
	M14						
	M15						
No activation response	M1						
	M6						
	M7						
	M10						
	M11						
	M12						
	M13						

**Table 3:** Changes in autofluorescence lifetime parameters of NAD(P)H α_1_ and τ_2_ in the patient lymphocytes on days 2 and 7 after in vitro anti-PD-1 or a combination of anti-PD-1 and anti-CTLA-4 treatment in comparison with the corresponding untreated controls. The color of the square indicates, respectively, a statistically significant (*p* ≤ 0.05, Student’s *t*-test) increase (green) or decrease (red) in a fluorescence lifetime parameter value compared to the corresponding untreated control. The colorless squares indicate no significant difference.

	Sample Code	Day 2		Day 7	
		α_1_	τ_2_	α_1_	τ_2_
Response	P2				
	P4				
	P7				
	P12				
	P13				
	P15				
	P16				
	P17				
	P18				
	P23				
Stable disease	P1				
	P8				
	P10				
	P19				
	P24				
Progressive disease	P6				
	P9				
	P14				
	P20				
	P21				
	P22				
	P26				

## Data Availability

Data are contained within the article.

## Supplementary Materials

The following supporting information can be downloaded at https://www.mdpi.com/article/10.3390/biomedicines14051049/s1: Supplementary Figure S1. Confocal fluorescence microscopy of a suspension of freshly isolated peripheral lymphocytes from a melanoma patient, labeled with (A) CD3-APC, (C) CD19-A647 antibody, and the overlay of the corresponding channels onto the brightfield images (B, D). Dashed outlines indicate examples of the same cells in the fluorescence channel and in the overlay on the brightfield image. Supplementary Table S1. Flow cytometry data on expression of CD3+ and CD19+ in the live human PBMC cell fraction after 2 and 7 days of culturing in the presence of IL-2; Supplementary Table S2. Primer sequences; Supplementary Table S3. Flow cytometry data on expression of the activation markers CD25 and CD69 in live mouse CD4+ Th and CD8+ T-lymphocytes after 2, 7 and 14 days of culturing in the presence of anti-CTLA-4 antibody; Supplementary Table S4. FLIM parameters of NAD(P)H in the mouse lymphocytes after 2, 7 and 14 days of culturing in the presence of anti-CTLA-4 antibody; Supplementary Table S5. Changes in autofluorescence lifetime parameters of NAD(P)H τ_m_ and τ_1_ in the mouse lymphocytes on days 2, 7 and 14 of in vitro anti-CTLA-4 treatment in comparison with the corresponding untreated controls. The color of the square indicates, respectively, a statistically significant (*p* ≤ 0.05, Student’s *t*-test) increase (green) or decrease (red) in a fluorescence lifetime parameter value compared to the corresponding untreated control. The colorless squares indicate no significant difference; Supplementary Table S6. FLIM parameters of NAD(P)H in patient lymphocytes after 2 and 7 days of culturing in the presence of anti-PD-1 or anti-PD-1 + anti-CTLA-4 antibodies; Supplementary Table S7. Changes in autofluorescence lifetime parameters of NAD(P)H τ_m_ and τ_1_ in the patient lymphocytes on days 2 and 7 of in vitro anti-PD-1 or a combination of anti-PD-1 and anti-CTLA-4 treatment in comparison with the corresponding untreated controls. The color of the square indicates, respectively, a statistically significant (*p* ≤ 0.05, Student’s *t*-test) increase (green) or decrease (red) in a fluorescence lifetime parameter value compared to the corresponding untreated control. The colorless squares indicate no significant difference.

## Abbreviations

The following abbreviations are used in this manuscript:

ICI	immune checkpoint inhibitor
FLIM	fluorescence lifetime imaging
NAD(P)H	nicotinamide adenine dinucleotide (phosphate)
CTLA-4	cytotoxic T-lymphocyte-associated antigen-4
PD-1/PD-L1	programmed cell death protein-1/ligand1
TMB	tumor mutational burden
MSI-H/dMMR	microsatellite instability/mismatch repair deficiency
FAO	fatty acid oxidation
RT-qPCR	reverse transcription quantitative polymerase chain reaction
RPMI	Roswell Park Memorial Institute
HEPES	4-(2-hydroxyethyl)-1-piperazineethanesulfonic acid
PBS	phosphate buffered saline
DMEM	Dulbecco’s Modified Eagle’s Medium
iRECIST	Immune Response Evaluation Criteria in Solid Tumors
iPR	immune partial response
iCR	immune complete response
iSD	immune stable disease
iCPD	immune confirmed progressive disease
iUPD	immune unconfirmed progressive disease
cTNM	clinical Tumor–Node–Metastasis staging
pTNM	pathological Tumor–Node–Metastasis staging
TCA cycle	tricarboxylic acid cycle
OXPHOS	oxidative phosphorylation
PPP	pentose phosphate pathway
G6PD	glucose-6-phosphate dehydrogenase
V(D)J	Variable (Diversity) Joining
PBMCs	peripheral blood mononuclear cells
