# Supplementary material for: Prediction of Patients’ Response to Immune Checkpoint Inhibitors Using Fluorescence Lifetime Imaging of Lymphocytes
Source: Biomedicines. 2026 May 5;14(5):1049. doi: 10.3390/biomedicines14051049 (PMC13204436; doi:10.3390/biomedicines14051049)
Supplement: Supplementary file 1 [file biomedicines-14-01049-s001.zip › biomedicines-4270493-supplementary.pdf]

2. Supplementary Materials and Methods

2.1 Experimental Design

Tumor spheroid preparation

To obtain 3D tumor spheroids, B16F0 melanoma cells were seeded at a concentration of 3 $\times 10^2$ cells per well in 96-well ultra-low attachment round-bottom plates (Corning, Glendale, AZ, USA) and cultured for 4 days in RPMI-1640 medium with L-glutamine (Roswell Park Memorial Institute 1640 medium). Spheroid morphology was evaluated using a DM IL LED inverted microscope (Leica Microsystems, Wetzlar, Germany). Then the tumor spheroids were gently aspirated using a cut pipette tip and transferred into 48-well flat-bottom plates (1-2 spheroids per well). After 24 hours of spheroid attachment to the plastic surface, immune cells were added.

2.2 Patient Blood Samples

Inclusion Criteria:

1. Signed informed consent.
2. Presence of histologically confirmed, treatment-naïve, unresectable, or metastatic cutaneous melanoma.
3. Presence of at least one measurable target lesion according to RECIST 1.1 criteria.
4. Planned first-line therapy with anti-PD-1 and/or anti-CTLA-4 antibodies.
5. ECOG performance status of 0–2.

Non-inclusion Criteria:

1. Prior systemic anticancer chemotherapy, immunotherapy, or treatment with immunosuppressive drugs within 5 years before study initiation.
2. Surgical treatment within 28 days or radiotherapy within 14 days prior to study initiation.
3. Acute infectious diseases or reactivation of chronic infections within 28 days prior to initiation.
4. Presence of systemic autoimmune diseases, other malignancies, or other severe pathologies.
5. HIV-positive status.
6. Any other significant diseases or conditions that, in the investigator’s opinion, could negatively affect the patient’s well-being or participation in the study, or distort the assessment of the results.

Exclusion Criteria (during the study):

1. Subsequent patient refusal to participate in the study.
2. Inability to assess the clinical outcome according to iRECIST due to treatment termination for any reason, inability to perform clinical evaluation due to the patient's health status, loss to follow-up, or other causes.
3. Occurrence of other severe diseases (excluding treatment-related adverse events, rAEs/irAEs) that could influence the clinical outcome.
4. The need for concurrent therapy with an effect on the immune system, excluding the use of another checkpoint inhibitor (anti-PD-1 or anti-CTLA-4) or their combination.

2.4 Lymphocyte Isolation and Culturing

To identify immune cell populations, patient lymphocytes were stained with antibodies to CD3 and CD19 (BD Biosciences, San Jose, CA, USA). The gating strategy was as follows: The lymphocyte population was first identified based on forward and side scatter (FSC/SSC) characteristics to exclude cell debris. Then the identification of T-cells (CD3+), B-cells (CD19+), and the non-T/non-B-cell fraction (CD3–/CD19–) was performed. Quantitative analysis was conducted on day 2 and day 7 of cultivation (Supplementary Table S1). Data were collected using a BD FACSAria III cell sorter (BD Biosciences, San Jose, CA, USA) and processed in FlowJo v10.5.3 (BD/Treestar, Ashland, OR, USA), following the established protocol [25].

Supplementary Table S1. Flow cytometry data on expression of CD3+ and CD19+ in the live human PBMC cell fraction after 2 and 7 days of culturing in the presence of IL-2.

Immune Cell Population	Day 2 (%, range)	Day 7 (%, range)

CD3+	72.4 – 85.4	85.2 – 94.8
CD19+	2.3 – 8.2	0.5 – 5.1
CD3−CD19−	5.4 – 13.8	3.5 – 7.4

2.6 FLIM of NAD(P)H

FLIM of NAD(P)H was carried out using an LSM 880 laser scanning confocal microscope (Carl Zeiss, Germany) equipped with an FLIM module Simple Tau 152 TCSPC (Becker & Hickl GmbH, Berlin, Germany) and a hybrid detector HPM-100-40 (Becker and Hickl GmbH, Berlin, Germany). A femtosecond Ti:Sapphire laser (80 MHz, 140 fs) was used for two-photon excitation of NAD(P)H fluorescence at 750 nm. Fluorescence of NAD(P)H was detected at 450–490 nm by a combination of a 490LP dichroic mirror and a ET475/50 bandpass filter (Chroma, US). The average laser power at the samples was about 6 mW. A C Plan-Apochromat 40×/1.3 NA Oil DIC objective was used for image acquisition. The field of view was 213 × 213 μm (512 × 512 pixels). Acquisition time of the images was 60 s. During FLIM image acquisition, the cells were maintained in the stage top incubator at 37°C and 5% CO2.

FLIM images were processed in SPCImage 8.3 software (Becker & Hickl GmbH, Germany). On average 5000-10000 photons were collected per decay curve at binning factor 3. Fitting was performed using a bi-exponential decay model using the weighted least square algorithm. The goodness of fit χ_2 was 0.8-1.2. The values of the short and long components of the lifetimes (τ_1 and τ_2) and their relative contributions (α_1 and α_2, $\alpha_1 + \alpha_2 = 100\%$) were obtained, which correspond to the free and protein-bound forms of the NAD(P)H cofactor, respectively. The weighted average (mean) lifetime was calculated as $\tau_m = (\alpha_1 * \tau_1 + \alpha_2 * \tau_2)/(\alpha_1 + \alpha_2)$. Fluorescence lifetimes were analyzed in cell cytoplasm by manual selection of the maximal area of cytoplasm as a region of interest in each individual cell. For each sample, FLIM images were acquired from 2-3 fields of view, with a total number of cells used for calculations of 30-40.

To ensure the accuracy of our cell selection, we performed preliminary experiments using confocal fluorescence microscopy with CD3 (T-cell) and CD19 (B-cell) staining according to the above protocol.

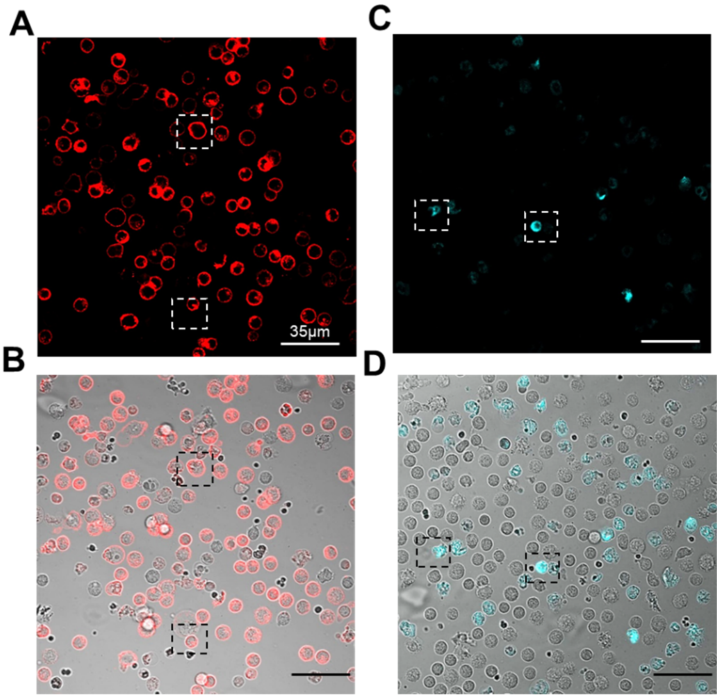

Supplementary Figure S1. Confocal fluorescence microscopy of a suspension of freshly isolated peripheral lymphocytes from a melanoma patient, labeled with (A) CD3-APC and (C) CD19-A647 antibody, and the overlay of the corresponding channels onto the brightfield images (B, D). Dashed outlines indicate examples of the same cells in the fluorescence channel and in the overlay on the brightfield image.

2.8 RT-qPCR

Supplementary Table S2. Primer sequences.

Primer Target	Primer Sequence
ACACA	F: TTCACTCCACCTTGTCAGCGGA
	R: GTCAGAGAAGCAGCCCATCACT
ACADM	F: TGGATAACCAACGGAGGAAAAG
	R: CTGGGGTATCTGCTTCCACA
ATP5F1B	F: AGTCTCCACCCGGACTACG
	R: CGCATAGTCCCTGACAGGATG
ELF1	F: GGATGAACGACAGCTTGGTG
	R: ATAGGGCCAGGGGAATCCAT
G6PD	F: TAGGCTGGAACCGCATCATC
	R: CCTGTTGGCAAATCTCAGCAC
HADHA	F: TTCAGCAACTTGACTGGGCA
	R: CAGTGATCTGGAATCACCGCT
HK1	F: CACCTGTGAGGTTGGACTCA
	R: CCACCATCTCCACGTTCTTC
HK2	F: GAGTTTGACCTGGATGTGGTTGC
	R: CCTCCATGTAGCAGGCATTGCT
LDHA	F: ACGTGCATTCCCGATTCCT
	R: AACAGCACCAACCCCAACAA
NDUFA6	F: GACGGGATAAAGTCCGAGAAATG
	R: TTCATGGAAGAACCGCATAACA
NDUFS1	F: AGTCTGTCATGGTGGAACCG
	R: CATCACACCTTCCCTGGCTT
OGDH	F: GAGCCGGAGACAGGCAGTTG
	R: CAGTCTGGGAAGCCGTCAAT
POLR2A	F: CCTGCAGACCGGCTATAAGG
	R: TCATCCCCGTCAAAGTCTGC
PDK1	F: CATGTCACGCTGGGTAATGAGG
	R: CTCAACACGAGGTCTTGGTGCA
TFB2M	F: CAGTTCCTTGGACAGCAGACAT
	R: GTGCCCTTTTCTCACCTCTACT

3. Supplementary Results

Supplementary Table S3. Flow cytometry data on expression of the activation markers CD25 and CD69 in live mouse CD4+ Th and CD8+ T-lymphocytes after 2, 7 and 14 days of culturing in the presence of anti-CTLA-4 antibody.

Sample code	Treatment		CD8+		CD4+	
			CD25+, %	CD69+, %	CD25+, %	CD69+, %
M1	2 days	no treatment	30.0±2.0	22.5±2.0	9.1±1.0	4.3±1.0
		anti-CTLA-4	30.7±2.0	23.0±2.0	9.3±1.0	4.6±1.0
	7 days	no treatment	40.1±2.0	38.7±2.0	10.5±1.0	3.4±1.0
		anti-CTLA-4	41.3±2.0	37.8±2.0	10.4±1.0	3.4±1.0
	14 days	no treatment	35.6±3.0	36.8±2.0	7.3±1.0	2.9±1.0
		anti-CTLA-4	28.4±3.0*	30.7±2.0*	5.1±1.0*	3.3±1.0
M2	2 days	no treatment	31.6±2.0	21.7±2.0	9.7±1.0	5.0±1.0
		anti-CTLA-4	32.5±2.0	21.3±2.0	10.4±1.0	5.1±1.0
	7 days	no treatment	46.7±2.0	45.6±2.0	9.0±1.0	3.0±1.0
		anti-CTLA-4	48.1±2.0	47.1±2.0	9.6±1.0	2.9±1.0
	14 days	no treatment	39.7±2.0	43.3±3.0	7.3±1.0	1.9±1.0

		anti-CTLA-4	45.8±3.0*	49.3±3.0*	7.1±1.0	2.0±1.0
M3	2 days	no treatment	30.2±2.0	23.6±2.0	15.4±1.0	5.8±1.0
		anti-CTLA-4	31.3±2.0	22.1±2.0	14.1±1.0	5.5±1.0
	7 days	no treatment	42.2±2.0	43.6±2.0	13.6±1.0	4.8±1.0
		anti-CTLA-4	45.6±2.0*	46.8±2.0*	13.5±1.0	4.5±1.0
	14 days	no treatment	38.2±2.0	42.4±2.0	6.2±1.0	2.1±1.0
		anti-CTLA-4	43.8±2.0*	48.9±2.0*	6.1±1.0	1.8±1.0
M4	2 days	no treatment	39.4±2.0	27.9±2.0	15.8±1.0	6.9±1.0
		anti-CTLA-4	38.1±2.0	28.5±2.0	15.9±1.0	6.6±1.0
	7 days	no treatment	50.0±2.0	49.8±2.0	14.0±1.0	5.4±1.0
		anti-CTLA-4	50.0±2.0	49.4±2.0	13.0±1.0	4.5±1.0
	14 days	no treatment	49.1±2.0	50.0±3.0	8.3±1.0	3.5±1.0
		anti-CTLA-4	56.9±3.0*	59.2±3.0*	12.9±1.0*	5.4±1.0*
M5	2 days	no treatment	31.2±2.0	20.9±2.0	12.5±1.0	5.7±1.0
		anti-CTLA-4	38.0±2.0*	24.2±2.0*	12.6±1.0	6.0±1.0
	7 days	no treatment	38.7±2.0	37.4±2.0	10.5±1.0	4.8±1.0
		anti-CTLA-4	43.3±2.0*	42.3±2.0*	13.5±1.0*	5.3±1.0
	14 days	no treatment	31.1±3.0	33.6±2.0	9.0±1.0	5.1±1.0
		anti-CTLA-4	37.7±2.0*	39.8±3.0*	12.1±1.0*	7.9±1.0*
M6	2 days	no treatment	23.7±2.0	17.5±2.0	9.0±1.0	4.0±1.0
		anti-CTLA-4	23.6±2.0	15.8±2.0	9.1±1.0	4.0±1.0
	7 days	no treatment	32.2±2.0	30.0±2.0	9.5±1.0	4.4±1.0
		anti-CTLA-4	33.1±2.0	30.6±2.0	9.3±1.0	4.0±1.0
	14 days	no treatment	31.2±2.0	32.2±2.0	8.5±1.0	6.6±1.0
		anti-CTLA-4	25.6±3.0*	26.3±2.0*	8.7±1.0	6.5±1.0
M7	2 days	no treatment	37.3±2.0	28.9±2.0	14.6±1.0	4.9±1.0
		anti-CTLA-4	36.7±2.0	28.4±2.0	14.8±1.0	5.1±1.0
	7 days	no treatment	41.8±2.0	42.3±2.0	12.5±1.0	3±1.0
		anti-CTLA-4	41.1±2.0	42.3±2.0	12.5±1.0	2.9±1.0
	14 days	no treatment	32.4±2.0	37.0±2.0	4.6±1.0	2.9±1.0
		anti-CTLA-4	25.2±3.0*	29.2±3.0*	2.6±1.0*	1.0±1.0*
M8	2 days	no treatment	27.1±2.0	18.5±2.0	11.4±1.0	3.2±1.0
		anti-CTLA-4	28.3±2.0	17.9±2.0	12.8±1.0	3.9±1.0
	7 days	no treatment	28.2±2.0	29.4±2.0	13.5±1.0	2.5±1.0
		anti-CTLA-4	31.9±2.0*	31.3±2.0*	15.5±1.0*	3.7±1.0
	14 days	no treatment	27.1±3.0	17.0±2.0	15.1±2.0	2.9±1.0
		anti-CTLA-4	33.8±2.0*	22.7±2.0*	18.8±1.0*	3.8±1.0
M9	2 days	no treatment	20.8±2.0	19.4±2.0	13.3±1.0	2.9±1.0
		anti-CTLA-4	22.6±2.0	21.3±2.0*	13.6±1.0	3.2±1.0
	7 days	no treatment	31.1±2.0	25.7±2.0	14.4±1.0	3.3±1.0
		anti-CTLA-4	36.7±3.0*	29.3±2.0*	14.6±1.0	5.0±1.0
	14 days	no treatment	30.5±3.0	20.1±3.0	15.0±2.0	4.1±1.0
		anti-CTLA-4	37.2±2.0*	26.1±2.0*	20.8±2.0*	4.8±1.0
M10	2 days	no treatment	31.3±2.0	19.4±2.0	7.2±1.0	4.6±1.0
		anti-CTLA-4	30.6±2.0	18.8±2.0	7.9±1.0	4.1±1.0
	7 days	no treatment	36.4±2.0	22.5±2.0*	16.7±1.0	4.6±1.0
		anti-CTLA-4	35.9±2.0	19.3±2.0	15.5±1.0	4.2±1.0
	14 days	no treatment	22.8±3.0	23.5±2.0	3.1±2.0	1.5±1.0
		anti-CTLA-4	22.1±2.0	22.8±3.0	2.9±1.0	1.2±1.0
M11	2 days	no treatment	28.4±2.0	18.6±2.0	3.5±1.0	2.4±1.0
		anti-CTLA-4	28.9±2.0	17.9±2.0	3.4±1.0	1.9±1.0
	7 days	no treatment	28.9±2.0	20.9±2.0	14.3±1.0	6.1±1.0

		anti-CTLA-4	28.4±1.0	19.2±1.0	14.7±1.0	6.4±1.0
	14 days	no treatment	25.4±3.0	23.7±2.0	4.2±1.5	1.8±1.0
		anti-CTLA-4	23.6±2.0	19.3±3.0*	4.3±1.0	1.9±1.0
M12	2 days	no treatment	30.0±2.0	28.4±2.0	3.1±1.0	5.5±1.0
		anti-CTLA-4	28.3±2.0	27.3±2.0	2.9±1.0	5.9±1.0
	7 days	no treatment	31.1±2.0	32.7±2.0	6.2±1.0	6.1±1.0
		anti-CTLA-4	28.8±2.0	24.0±2.0*	5.0±1.0	7.2±1.0
	14 days	no treatment	30.0±2.0	31.4±2.0	5.1±1.0	6.5±1.0
		anti-CTLA-4	25.3±2.0*	23.3±3.0*	4.2±1.0	6.9±1.0
M13	2 days	no treatment	22.9±2.0	19.6±2.0	4.1±1.0	3.0±1.0
		anti-CTLA-4	24.1±2.0	18.6±2.0	2.8±1.0	2.5±1.0
	7 days	no treatment	22.7±2.0	27.8±2.0	5.3±1.0	3.0±1.0
		anti-CTLA-4	22.0±2.0	27.0±2.0	3.5±1.0	2.0±1.0
	14 days	no treatment	21.9±3.0	25.3±2.0	4.1±1.0	2.0±1.0
		anti-CTLA-4	20.1±3.0	24.6±3.0	3.8±1.0	1.5±1.0
M14	2 days	no treatment	29.1±2.0	19.0±2.0	6.7±1.0	2.9±1.0
		anti-CTLA-4	34.8±3.0*	22.3±2.0*	6.8±1.0	3.8±1.0
	7 days	no treatment	31.1±2.0	19±2.0	8.1±1.0	5.9±1.0
		anti-CTLA-4	35.8±3.0*	22.5±2.0*	9.1±1.0	5.8±1.0
	14 days	no treatment	30.1±3.0	19.0±3.0	10.1±1.0	6.9±1.0
		anti-CTLA-4	38.8±3.0*	26.7±2.0*	12.8±1.0*	7.8±1.0
M15	2 days	no treatment	35.4±2.0	30.2±2.0	4.0±0.9	1.9±1.0
		anti-CTLA-4	38.1±2.0	33.2±2.0	4.1±1.0	3.1±1.0
	7 days	no treatment	37.1±2.0	32.6±2.0	6.0±0.9	3.1±1.0
		anti-CTLA-4	42.7±2.0*	35.1±2.0	7.1±1.0	4.8±1.0
	14 days	no treatment	36.1±3.0	31.6±3.0	8.0±0.9	4.1±1.0
		anti-CTLA-4	44.7±3.0*	37.8±3.0*	10.1±1.0*	6.1±1.0

* Statistically significant difference compared to the corresponding group "no treatment", $p \leq 0{,}05$, Student's t-test. Data are presented as means ± SEM.

Supplementary Table S4. FLIM parameters of NAD(P)H in the mouse lymphocytes after 2, 7 and 14 days of culturing in the presence of anti-CTLA-4 antibody.

Sample code	Treatment		FLIM parameters of NAD(P)H				
			τ_m	τ_1	τ_2	α_1	α_2
M1	2 days	no treatment	1.28±0.03	0.43±0.04	2.89±0.07	65.7±0.9	36.5±0.9
		anti-CTLA-4	1.30±0.03	0.45±0.04	2.71±0.07	62.5±0.9*	40.5±0.9*
	7 days	no treatment	1.33±0.03	0.48±0.04	3.06±0.04	67.1±0.9	32.92±0.9
		anti-CTLA-4	1.13±0.04*	0.38±0.04*	2.41±0.08*	63.2±0.5*	36.8±0.5*
	14 days	no treatment	1.32±0.02	0.48±0.04	3.14±0.06	67.9±0.5	32.1±0.5
		anti-CTLA-4	1.18±0.03*	0.40±0.04*	2.78±0.08*	66.6±0.5	33.39±0.5
M2	2 days	no treatment	1.24±0.02	0.57±0.02	2.53±0.06	65.4±1.0	34.6±1.0
		anti-CTLA-4	1.22±0.03	0.52±0.02	2.53±0.06	65.0±1.0	35.0±1.0
	7 days	no treatment	1.37±0.04	0.50±0.02	2.91±0.06	63.9±0.8	36.1±0.8
		anti-CTLA-4	1.44±0.04*	0.54±0.02	3.39±0.07*	68.3±0.8*	31.7±0.8*
	14 days	no treatment	1.22±0.04	0.39±0.02	2.80±0.03	65.4±0.6	34.6±0.6
		anti-CTLA-4	1.29±0.04	0.43±0.02	3.21±0.03*	69.1±0.5*	30.9±0.5*
M3	2 days	no treatment	1.17±0.04	0.48±0.03	2.39±0.08	64.0±0.8	36.0±0.8
		anti-CTLA-4	1.38±0.05*	0.54±0.03	3.05±0.07*	66.7±0.9*	33.35±0.9*
	7 days	no treatment	1.28±0.03	0.55±0.01	2.94±0.04	69.5±0.8	30.5±0.8
		anti-CTLA-4	1.31±0.03	0.59±0.01	3.19±0.04*	72.5±0.7*	27.5±0.7*
	14 days	no treatment	1.17±0.03	0.42±0.01	2.85±0.05	69.0±0.7	31.0±0.7
		anti-CTLA-4	1.29±0.02*	0.48±0.01*	3.14±0.05*	69.5±0.63	30.5±0.6

M4	2 days	no treatment	1.21±0.03	0.53±0.02	2.80±0.05	70.0±0.9	30.0±0.9
		anti-CTLA-4	1.31±0.03*	0.57±0.02	3.01±0.05*	69.6±0.9	30.4±0.9
	7 days	no treatment	1.28±0.04	0.53±0.01	2.86±0.06	67.8±0.8	32.2±0.8
		anti-CTLA-4	1.36±0.03*	0.51±0.01	3.16±0.04*	67.9±0.5	32.1±0.5
	14 days	no treatment	1.11±0.03	0.43±0.03	2.38±0.07	64.5±0.8	35.5±0.8
		anti-CTLA-4	1.24±0.03*	0.48±0.03	2.85±0.06*	67.5±0.6*	32.50±0.6*
M5	2 days	no treatment	1.25±0.03	0.52±0.02	2.95±0.08	69.1±0.8	30.9±0.8
		anti-CTLA-4	1.49±0.03*	0.66±0.01*	3.66±0.05*	72.2±0.7*	27.8±0.7*
	7 days	no treatment	1.54±0.03	0.53±0.02	3.65±0.05	67.7±0.5	32.3±0.5
		anti-CTLA-4	1.52±0.03	0.59±0.01	3.66±0.04	69.7±0.7*	30.3±0.7*
	14 days	no treatment	1.49±0.03	0.58±0.01	3.62±0.05	70.1±0.4	29.9±0.4
		anti-CTLA-4	1.39±0.02*	0.56±0.01	3.69±0.04	73.3±0.4*	26.7±0.4*
M6	2 days	no treatment	1.23±0.04	0.55±0.02	2.72±0.04	67.9±0.8	32.1±0.8
		anti-CTLA-4	1.13±0.04*	0.50±0.02	2.56±0.04*	68.8±0.5	31.2±0.5
	7 days	no treatment	1.48±0.03	0.64±0.01	3.30±0.04	68.5±0.5	31.5±0.5
		anti-CTLA-4	1.37±0.02*	0.58±0.01*	3.11±0.03*	68.8±0.6	31.3±0.6
	14 days	no treatment	1.29±0.03	0.48±0.01	3.07±0.07	68.6±0.5	31.4±0.5
		anti-CTLA-4	1.16±0.02*	0.46±0.01*	2.35±0.05*	62.8±0.8*	37.2±0.8*
M7	2 days	no treatment	1.20±0.02	0.53±0.01	2.86±0.05	71.2±0.7	28.8±0.7
		anti-CTLA-4	1.18±0.02	0.51±0.01	2.58±0.04*	67.9±0.5*	32.1±0.5*
	7 days	no treatment	1.52±0.03	0.58±0.02	3.61±0.04	69.0±0.7	31.0±0.7
		anti-CTLA-4	1.26±0.05*	0.41±0.01*	2.68±0.09*	62.6±0.7*	37.4±0.7*
	14 days	no treatment	1.34±0.03	0.45±0.03	3.37±0.04	69.5±0.7	30.5±0.7
		anti-CTLA-4	1.13±0.04*	0.38±0.03*	2.45±0.04*	63.8±0.8*	36.2±0.7*
M8	2 days	no treatment	1.25±0.03	0.56±0.03	2.61±0.05	66.4±0.6	33.6±0.6
		anti-CTLA-4	1.28±0.03	0.55±0.03	2.71±0.06	66.0±0.6	34.0±0.6
	7 days	no treatment	1.08±0.04	0.47±0.02	2.52±0.05	70.3±0.9	29.7±0.9
		anti-CTLA-4	1.01±0.04	0.50±0.02	2.62±0.05	76.2±0.8*	23.8±0.8*
	14 days	no treatment	1.10±0.04	0.46±0.03	2.79±0.06	72.9±0.8	27.1±0.8
		anti-CTLA-4	1.12±0.04	0.54±0.03	2.88±0.05	75.4±0.8*	24.6±0.8*
M9	2 days	no treatment	1.17±0.03	0.55±0.02	2.50±0.05	68.0±0.7	32.00±0.7
		anti-CTLA-4	1.12±0.03	0.56±0.02	2.56±0.05	71.7±0.7*	28.3±0.7
	7 days	no treatment	1.12±0.06	0.44±0.02	2.62±0.09	69.0±0.8	31.0±0.8
		anti-CTLA-4	0.98±0.04	0.45±0.02	2.45±0.08	73.4±1.0*	26.6±1.0*
	14 days	no treatment	1.17±0.03	0.54±0.02	2.70±0.06	70.5±0.7	29.5±0.7
		anti-CTLA-4	1.18±0.03	0.54±0.02	2.95±0.06*	73.6±0.7*	26.4±0.7*
M10	2 days	no treatment	1.30±0.03	0.56±0.03	2.76±0.06	66.1±0.7	33.9±0.7
		anti-CTLA-4	1.26±0.03	0.53±0.03	2.78±0.06	67.7±0.7	32.3±0.7
	7 days	no treatment	1.10±0.04	0.46±0.02	2.56±0.04	69.5±0.6	30.5±0.6
		anti-CTLA-4	1.14±0.03	0.43±0.01	2.61±0.06	67.3±0.67*	32.7±0.67*
	14days	no treatment	1.27±0.04	0.54±0.03	2.91±0.06	69.4±0.7	30.6±0.7
		anti-CTLA-4	1.28±0.04	0.58±0.03	2.72±0.06*	67.9±0.7*	32.1±0.7
M11	2 days	no treatment	1.42±0.03	0.62±0.04	2.73±0.05	62.1±0.8	37.9±0.8
		anti-CTLA-4	1.39±0.03*	0.60±0.04*	2.7±0.05	62.3±0.8	37.7±0.8
	7 days	no treatment	1.31±0.06	0.49±0.02	2.65±0.09	62.0±1.3	38.0±1.3
		anti-CTLA-4	1.35±0.06	0.50±0.02	2.78±0.09	62.6±1.3	37.4±1.3
	14 days	no treatment	1.20±0.03	0.51±0.02	2.52±0.04	65.6±0.8	34.4±0.8
		anti-CTLA-4	1.11±0.03*	0.55±0.02	2.19±0.04*	65.4±0.8	34.6±0.8
M12	2 days	no treatment	1.12±0.04	0.49±0.03	2.43±0.05	67.6±0.8	32.4±0.8
		anti-CTLA-4	1.10±0.04	0.44±0.03	2.38±0.05	66.2±0.8	33.8±0.8
	7 days	no treatment	1.24±0.03	0.50±0.01	2.79±0.04	67.4±0.5	32.6±0.5
		anti-CTLA-4	1.21±0.03	0.43±0.01	2.71±0.04	65.7±0.5*	34.3±0.5*

	14 days	no treatment	1.23±0.05	0.40±0.04	2.96±0.06	67.7±0.8	32.3±0.8
		anti-CTLA-4	1.01±0.05*	0.33±0.04*	2.20±0.06*	63.4±0.8*	36.6±0.8*
M13	2 days	no treatment	1.19±0.04	0.51±0.03	2.49±0.05	65.6±0.6	34.4±0.6
		anti-CTLA-4	1.15±0.04	0.47±0.03	2.48±0.05	66.0±0.6	34.0±0.6
	7 days	no treatment	1.30±0.03	0.50±0.01	2.82±0.04	65.4±0.6	34.6±0.6
		anti-CTLA-4	1.30±0.03	0.47±0.01	2.79±0.04	64.4±0.5	35.6±0.5
	14 days	no treatment	1.10±0.04	0.40±0.02	2.56±0.04	67.7±0.6	32.3±0.6
		anti-CTLA-4	1.15±0.04	0.45±0.02	2.51±0.04	65.9±0.6*	34.1±0.6
M14	2 days	no treatment	1.15±0.05	0.49±0.04	2.56±0.03	67.9±0.7	32.1±0.7
		anti-CTLA-4	1.25±0.05*	0.60±0.04*	2.76±0.03*	69.8±0.7	30.2±0.7
	7 days	no treatment	1.27±0.03	0.47±0.01	2.79±0.04	65.6±0.6	34.4±0.6
		anti-CTLA-4	1.37±0.04	0.55±0.02*	3.12±0.06*	68.1±0.5*	31.9±0.5*
	14 days	no treatment	1.30±0.04	0.59±0.02	3.02±0.05	70.4±0.8	29.6±0.8
		anti-CTLA-4	1.40±0.04*	0.68±0.02*	3.30±0.05*	72.5±0.8*	27.5±0.8*
M15	2 days	no treatment	1.34±0.03	0.67±0.03	2.97±0.05	70.8±0.9	29.2±0.9
		anti-CTLA-4	1.34±0.03	0.59±0.03	3.12±0.05	70.4±0.9	29.6±0.9
	7 days	no treatment	1.26±0.03	0.47±0.01	2.77±0.04	65.8±0.5	34.2±0.5
		anti-CTLA-4	1.45±0.04*	0.61±0.01*	3.21±0.06*	67.8±0.5*	32.2±0.5*
	14 days	no treatment	1.34±0.03	0.60±0.02	3.08±0.04	69.8±0.8	30.2±0.8
		anti-CTLA-4	1.43±0.03*	0.65±0.02	3.21±0.04*	72.3±0.8*	27.7±0.8*

* Statistically significant difference compared to the corresponding group "no treatment", $p \le 0,05$, Student's t-test. Data are presented as means ± SEM.

Supplementary Table S5. Changes in autofluorescence lifetime parameters of NAD(P)H τ_m and τ_1 in the mouse lymphocytes on days 2, 7 and 14 of in vitro anti-CTLA-4 treatment in comparison with the corresponding untreated controls. The color of the square indicates, respectively, a statistically significant (p ≤ 0.05, Student's t-test) increase (green) or decrease (red) in a fluorescence lifetime parameter value compared to the corresponding untreated control. The colorless squares indicate no significant difference.

	Sample code	Day 2		Day 7		Day 14	
		τ_m	τ_1	τ_m	τ_1	τ_m	τ_1
Activation response	M2						
	M3						
	M4						
	M5						
	M8						
	M9						
	M14						
	M15						
No activation response	M1						
	M6						
	M7						
	M10						
	M11						
	M12						
	M13						

Supplementary Table S6. FLIM parameters of NAD(P)H in patient lymphocytes after 2 and 7 days of culturing in the presence of anti-PD-1 or anti-PD-1 + anti-CTLA-4 antibodies.

Sample code	Treatment		FLIM parameters of NAD(P)H				
			τ_m	τ_1	τ_2	α_1	α_2
P1	2 days	no treatment	1.45±0.02	0.64±0.01	3.38±0.04	69.4±0.5	30.6±0.5
		anti-PD-1	1.47±0.01	0.65±0.01	3.42±0.03	69.0±0.7	31.0±0.7
	7 days	no treatment	1.39±0.04	0.57±0.02	3.19±0.07	67.1±1.0	32.9±1.0

		anti-PD-1	1.35±0.04	0.54±0.02	3.09±0.07	67.4±1.0	33.1±1.0
P2	2 days	no treatment	1.32±0.04	0.56±0.01	3.40±0.05	72.3±1.1	27.7±1.1
		anti-PD-1	1.42±0.02*	0.61±0.01*	3.70±0.04*	73.3±0.5	26.7±0.5
	7 days	no treatment	1.45±0.02	0.62±0.01	3.64±0.06	71.5±0.5	28.5±0.5
		anti-PD-1	1.44±0.02	0.65±0.01	3.84±0.05*	74.2±0.6*	25.8±0.6*
P4	2 days	no treatment	1.31±0.03	0.54±0.01	3.36±0.07	71.3±0.8	28.7±0.8
		anti-PD-1	1.30±0.03	0.58±0.02	3.31±0.05	71.7±0.5	27.5±0.7
	7 days	no treatment	1.29±0.04	0.49±0.02	2.92±0.07	65.5±0.9*	34.5±0.9*
		anti-PD-1	1.21±0.02	0.52±0.01	3.09±0.07	70.9±0.6*	29.1±0.6*
P6	2 days	no treatment	1.37±0.03	0.60±0.01	3.59±0.05	73.3±0.6	26.7±0.6
		anti-PD-1+ anti-CTLA-4	1.29±0.03*	0.55±0.01*	3.11±0.05*	70.4±1.3*	29.7±1.3*
	7 days	no treatment	1.40±0.05	0.56±0.01	3.45±0.09	69.9±1.2	30.1±1.2
		anti-PD-1+ anti-CTLA-4	1.33±0.04	0.51±0.01*	3.17±0.07*	69.0±1.0	31.6±1.2
P7	2 days	no treatment	1.32±0.02	0.58±0.01	3.39±0.06	72.6±0.8	27.4±0.8
		anti-PD-1	1.43±0.02*	0.63±0.01*	3.66±0.07*	72.8±0.8	27.3±0.8
	7 days	no treatment	1.43±0.03	0.61±0.01	3.62±0.05	72.3±0.8*	27.8±0.8*
		anti-PD-1	1.38±0.01	0.62±0.01	3.63±0.03	74.2±0.4*	25.8±0.4*
P8	2 days	no treatment	1.37±0.03	0.58±0.01	3.32±0.04	70.6±0.8	29.4±0.8
		anti-PD-1	1.28±0.02*	0.53±0.01*	3.06±0.05*	69.9±0.9	30.1±0.9
	7 days	no treatment	1.40±0.02	0.61±0.01	3.67±0.05	73.6±0.6	26.5±0.6
		anti-PD-1	1.44±0.03	0.62±0.01	3.58±0.07	71.5±0.7*	28.5±0.7*
P9	2 days	no treatment	1.33±0.04	0.60±0.02	3.29±0.11	72.0±0.5	28.0±0.5
		anti-PD-1	1.40±0.02	0.60±0.01	3.27±0.07	69.2±0.7*	30.8±0.7*
	7 days	no treatment	1.39±0.02	0.62±0.01	3.51±0.05	72.0±0.6	28.0±0.6
		anti-PD-1	1.35±0.03	0.57±0.01	3.29±0.08*	70.3±0.6*	29.7±0.6*
P10	2 days	no treatment	1.38±0.02	0.62±0.01	3.71±0.05	74.6±0.9	25.4±0.9
		anti-PD-1	1.35±0.04	0.56±0.01*	3.52±0.05*	72.7±0.8	27.3±0.8
	7 days	no treatment	1.41±0.02	0.64±0.01	3.61±0.05	73.1±0.6	26.9±0.6
		anti-PD-1	1.37±0.03	0.60±0.01	3.53±0.06	72.8±0.7	27.2±0.7
P12	2 days	no treatment	1.40±0.02	0.59±0.01	3.31±0.05	69.4±0.5	30.6±0.5
		anti-PD-1+ anti-CTLA-4	1.41±0.04	0.63±0.01*	3.54±0.06*	72.7±0.9*	27.4±0.9*
	7 days	no treatment	1.16±0.01	0.51±0.01	2.84±0.05	71.3±0.5	28.7±0.5
		anti-PD-1+ anti-CTLA-4	1.36±0.01*	0.61±0.01*	3.44±0.06*	72.3±0.5	27.7±0.5
P13	2 days	no treatment	1.35±0.02	0.60±0.01	3.83±0.03	76.2±0.5	23.8±0.5
		anti-PD-1	1.33±0.02	0.60±0.01	3.92±0.04	77.7±0.5*	22.3±0.5*
	7 days	no treatment	1.30±0.04	0.50±0.02	2.99±0.07	66.6±1.4	33.4±1.4
		anti-PD-1	1.26±0.03	0.51±0.01	3.18±0.04*	70.9±1.1*	29.1±1.1*
P14	2 days	no treatment	1.31±0.02	0.59±0.01	3.63±0.05	75.7±0.5	24.3±0.5
		anti-PD-1	1.29±0.03	0.59±0.01	3.35±0.05*	74.2±0.6	25.8±0.6
	7 days	no treatment	1.38±0.03	0.58±0.01	3.37±0.07	70.1±0.0	30.0±0.0
		anti-PD-1	1.39±0.03	0.53±0.01*	3.00±0.07*	65.1±1.0*	36.1±1.0*
P15	2 days	no treatment	1.04± 0.02	0.44 ±0.01	2.68±0.07	72.5±0.7	27.5±0.7
		anti-PD-1	1.12±0.02*	0.51±0.01*	2.94±0.06*	74.1±0.7	25.9±0.7
	7 days	no treatment	1.29±0.02	0.56±0.01	3.22±0.05	71.8±0.7	28.2±0.7
		anti-PD-1	1.32±0.02	0.59±0.01	3.45±0.04*	73.7±0.6*	26.3±0.6*
P16	2 days	no treatment	1.34±0.02	0.57±0.004	3.67±0.05	74.3±0.6	25.8±0.6
		anti-PD-1	1.40±0.03	0.59±0.01	3.70±0.04	72.9±1.1	27.1±1.1
	7 days	no treatment	1.43±0.02	0.61±0.01	3.43±0.08	70.0±0.7	30.0±0.7

		anti-PD-1	1.50±0.03	0.63±0.01	3.66±0.04*	70.4±0.7	29.6±0.7
P17	2 days	no treatment	1.38±0.02	0.64±0.01	3.32±0.06	71.4±0.8	28.7±0.8
		anti-PD-1	1.39±0.03	0.62±0.01	3.86±0.05*	75.8±0.8*	24.5±0.7*
	7 days	no treatment	1.47±0.03	0.63±0.01	3.84±0.04	73.6±0.5	26.4±0.5
		anti-PD-1	1.46±0.03	0.62±0.01	3.86±0.04	73.6±0.6	26.5±0.6
P18	2 days	no treatment	1.12±0.02	0.51±0.01	2.91±0.06	73.9±0.9	26.1±0.9
		anti-PD-1	1.42±0.02*	0.62±0.01*	3.93±0.04*	75.5±0.7*	24.5±0.7*
	7 days	no treatment	1.27±0.05	0.55±0.02	3.50±0.06	75.1±1.1	24.9±1.1
		anti-PD-1	1.36±0.04*	0.58±0.01	3.78±0.08*	75.2±0.8	24.8±0.8
P19	2 days	no treatment	1.31±0.02	0.59±0.01	3.33±0.06	73.1±0.7	27.0±0.7
		anti-PD-1	1.41±0.03*	0.63±0.01*	3.61±0.05*	73.1±1.0	26.9±1.0
	7 days	no treatment	1.54±0.04	0.63±0.01	4.01±0.05	72.8±0.8	27.3±0.8
		anti-PD-1	1.47±0.04	0.61±0.02	3.82±0.07*	72.8±1.0	27.2±1.0
P20	2 days	no treatment	1.28±0.02	0.53±0.01	3.31±0.06	72.3±0.6	27.7±0.6
		anti-PD-1	1.25±0.01	0.53±0.01	3.34±0.08	73.6±0.6	26.4±0.6
	7 days	no treatment	1.33±0.02	0.56±0.02	3.69±0.06	75.0±0.6	25.0±0.6
		anti-PD-1	1.30±0.02	0.59±0.02	3.39±0.05*	74.0±0.5	26.0±0.6
P21	2 days	no treatment	1.38±0.03	0.60±0.01	3.46±0.06	71.9±0.6	28.1±0.6
		anti-PD-1	1.35±0.02	0.59±0.01	3.37±0.05	71.4±0.8	28.6±0.8
	7 days	no treatment	1.65±0.02	0.69±0.01	4.13±0.04	71.7±0.6	28.3±0.6
		anti-PD-1	1.47±0.03*	0.61±0.01*	3.53±0.07*	69.9±0.5*	30.1±0.5*
P22	2 days	no treatment	1.21±0.01	0.53±0.01	2.88±0.04	70.4±0.5	29.6±0.5
		anti-PD-1	1.21±0.01	0.52±0.01	2.93±0.05	70.8±0.6	29.3±0.6
	7 days	no treatment	1.32±0.02	0.58±0.01	3.38±0.07	72.7±0.5	27.3±0.5
		anti-PD-1	1.26±0.02	0.55±0.01	3.09±0.05*	71.5±0.5	28.5±0.5
P23	2 days	no treatment	1.05±0.02	0.42±0.01	2.37±0.06	67.4±1.0	32.6±1.0
		anti-PD-1	1.03±0.02	0.45±0.01	2.56±0.07*	72.0±1.3*	28.1±1.3*
	7 days	no treatment	1.09±0.02	0.46±0.02	2.53±0.07	69.3±0.6	30.7±0.6
		anti-PD-1	1.19±0.03*	0.51±0.02	2.82±0.08*	70.3±0.6	29.7±0.6
P24	2 days	no treatment	1.18±0.02	0.50±0.01	2.76±0.05	69.8±0.5	30.2±0.5
		anti-PD-1	1.15±0.01	0.49±0.01	2.72±0.04	70.1±0.5	29.9±0.5
	7 days	no treatment	1.23±0.02	0.54±0.01	2.93±0.07	70.4±0.9	29.7±0.9
		anti-PD-1	1.12±0.02*	0.46±0.01*	2.61±0.04*	69.0±0.7	31.0±0.7
P26	2 days	no treatment	1.34±0.03	0.53±0.01	3.02±0.09	66.3±1.4	33.7±1.4
		anti-PD-1	1.20±0.02	0.48±0.01	2.79±0.05*	68.4±0.8	31.6±0.8
	7 days	no treatment	1.25±0.03	0.46±0.01	2.83±0.06	66.1±1.1	33.9±1.1
		anti-PD-1	1.38±0.03*	0.50±0.01	2.88±0.04	62.8±0.8*	37.2±0.8*

* Statistically significant difference compared to the corresponding group "no treatment", $p \leq 0{,}05$. Data are presented as means ± SEM.

Supplementary Table S7. Changes in autofluorescence lifetime parameters of NAD(P)H τ_m and τ_1 in the patient lymphocytes on days 2 and 7 of in vitro anti-PD-1 or a combination of anti-PD-1 and anti-CTLA-4 treatment in comparison with the corresponding untreated controls. The color of the square indicates, respectively, a statistically significant (p ≤ 0.05, Student's t-test) increase (green) or decrease (red) in a fluorescence lifetime parameter value compared to the corresponding untreated control. The colorless squares indicate no significant difference.

	**Sample code**	**Day 2**		**Day 7**	
		**τ_m**	**τ_1**	**τ_m**	**τ_1**
Response	P2				
	P4				
	P7				
	P12				
	P13				
	P15				

	P16				
	P17				
	P18				
	P23				
Stable disease	P1				
	P8				
	P10				
	P19				
	P24				
Progressive disease	P6				
	P9				
	P14				
	P20				
	P21				
	P22				
	P26				
